# Do Case Rates Affect Physicians' Clinical Practice in Radiation Oncology?: An Observational Study

**DOI:** 10.1371/journal.pone.0149449

**Published:** 2016-02-12

**Authors:** Bryan A. Loy, Clive I. Shkedy, Adam C. Powell, Laura E. Happe, Julie A. Royalty, Michael T. Miao, Gary L. Smith, James W. Long, Amit K. Gupta

**Affiliations:** 1 Humana Inc., Louisville, Kentucky, United States of America; 2 HealthHelp, LLC, Houston, Texas, United States of America; University of California, San Diego, UNITED STATES

## Abstract

Case rate payments combined with utilization monitoring may have the potential to improve the quality of care by reducing over and under-treatment. Thus, a national managed care organization introduced case rate payments at one multi-site radiation oncology provider while maintaining only fee-for-service payments at others. This study examined whether the introduction of the payment method had an effect on radiation fractions administered when compared to clinical guidelines. The number of fractions of radiation therapy delivered to patients with bone metastases, breast, lung, prostate, and skin cancer was assessed for concordance with clinical guidelines. The proportion of guideline-based care ascertained from the payer's claims database was compared before (2011) and after (2013) the payment method introduction using relative risks (RR). After the introduction of case rates, there were no significant changes in guideline-based care in breast, lung, and skin cancer; however, patients with bone metastases and prostate cancer were significantly more likely to have received guideline-based care (RR = 2.0 and 1.1, respectively, p<0.05). For the aggregate of all cancers, the under-treatment rate significantly declined (p = 0.008) from 4% to 0% after the introduction of case rate payments, while the over-treatment rate remained steady at 9%, with no significant change (p = 0.20). These findings suggest that the introduction of case rate payments did not adversely affect the rate of guideline-based care at the provider examined. Additional research is needed to isolate the effect of the payment model and assess implications in other populations.

## Introduction

Radiation oncology providers and health plans have historically engaged in fee-for-service contractual relationships where health plans reimburse physicians based on treatments administered. Under fee-for-service, radiation benefit managers (RBM) are sometimes employed to provide one-time authorizations for complete radiation oncology treatment plans. The authorization process aims to ensure radiation oncology treatment plans conform to guideline-based protocols, in order to avoid inappropriate radiation therapy.

There are several challenges with the fee-for-service payment methodology. Fee-for-service incents a higher volume of care by tying revenue to activity. Beyond incenting a higher volume of care, fee-for-service can result in unpredictable revenue streams for providers, including extended revenue cycle time and complicated accounts receivable analyses. Moreover, the administrative process requires prior authorization and coding of multiple services, both of which are burdensome on both providers and payers. Under fee-for-service as it is presently implemented, there are limited mechanisms for providers and payers to systematically measure and reward quality. Recognizing these challenges, the Centers for Medicare & Medicaid Services (CMS) has explored case rate payments for radiation therapy, yet case rate payments have not been incorporated into the Medicare Physician Fee Schedule to date. [1]

Case rate payments address many of the challenges associated with fee-for-service. In radiation oncology, case rate payments reduce the need to review the cost associated with the modality of treatment, thereby leaving the treatment plan decision with the treating physician and reducing the administrative burden. As the treating physician bears some of the cost of over-treatment, case rates may have the potential to reduce iatrogenic effects. Furthermore, it has been suggested that case rates have the potential to substantially reduce healthcare costs. [2] Contrary to fee-for-service arrangements, which can incentivize treatment in excess of clinical guidelines (over-treatment), case rate payments have the potential to encourage treatment below clinical guidelines (under-treatment), which can lead to cancer recurrence.

In August of 2012, Humana Inc., a national health insurance provider and 21st Century Oncology, a large for-profit radiation oncology multi-site provider, executed the first known contract for case rate payments in radiation oncology. The design of the payment model has been published previously; [3] yet there is inadequate evidence in the literature to determine whether case rates payments affect patient care in radiation oncology. Therefore, this study examined whether the introduction of the payment method had an effect on the quality of care, as measured by the radiation fractions administered, when compared to clinical guidelines.

## Materials and Methods

This retrospective study compared the proportion of patients receiving guideline-based care in 2011 under fee-for-service reimbursement and in 2013 after case rate payments were introduced. Humana’s administrative claims data were used for the analysis. Data from 2012 were not assessed since the payment system was introduced mid-year. The study population was comprised of individuals residing in 20 states (highest concentration in Arizona and Florida) with commercial or Medicare insurance provided by Humana. As this study was conducted as a part of Humana’s normal quality improvement operations, it did not meet the Department of Health and Human Service’s regulatory definition of research under 45 Code of Federal Regulations 46.102(d), and thus did not require Institutional Review Board approval. The authors have access to patient identifying information through the course of their daily job responsibilities and have accessed such data to complete this work.

The individual clinics run by the provider which treated the patients share common treatment protocols, and 51% of Humana members treated by them in 2013 were subject to case rate payments. A sub-analysis was conducted for the subset of patients covered by case rate payments only. For each cancer type, the change in the rate of guideline-based care between 2011 and 2013 in patients subject and not subject to case rate payments was compared using a chi-squared test. An unknown number of patients belonging to other health plans were also treated by the provider. As a result, the proportion of patients within each clinic and across the provider exposed to case rate payments is unknown. For the main analysis, all patients were grouped using an intent-to-treat approach, as providers are unlikely to be aware of the payment arrangements of individual patients.

Due to sample size limitations, only patients being treated for bone metastases, breast cancer, lung cancer, skin cancer, and prostate cancer were included in the analysis. Likewise, only patients treated with 2 dimensional 3 dimensional (2D3D) or intensity-modulated radiation therapy (IMRT) were included. The data were validated to ensure that none of the 2D3D patients had received an IMRT treatment plan (CPT 77301). Patients who initiated care after December 1^st^ or completed care before January 31^st^ were excluded, as these patients may have incomplete claims data due to changes in plan membership. It was not possible to determine which patients received palliative radiation, as clinical data were not examined.

The study defined the number of fractions of radiation considered to be guideline-based according to the National Comprehensive Cancer Network (NCCN) guidelines, American Society for Radiation Oncology (ASTRO) guidelines, and published literature **(Table 1)**. [4–9] The guidelines were validated by the Medical Director of Radiation Oncology at HealthHelp, LLC, the pre-authorization services company overseeing the patients, to ensure their consistency with the company’s practice and to convert the numbers of gray recommended in the literature into numbers of fractions. The levels of radiation used to determine what should constitute appropriate care were broad, as the guidelines may vary according to patient demographics, such as age.

**Table 1:** Number of fractions defined as guideline-based care.

	2D3D Range	IMRT Range
**Bone Metastases**	1 to 10	1 to 10
**Breast**	16 to 35	16 to 35
**Lung**	10 to 37	10 to 37
**Prostate**	25 to 39	25 to 45
**Skin**	5 to 33	5 to 35

During the conversion process, a fraction was considered to deliver between 180 and 200 centigray (cGy) of radiation, as that is the practice of the providers studied. To arrive at a conservative estimate of guideline-based care, the lower bound of guideline-based care was set as the number of 200 cGy fractions which would need to be delivered to achieve the minimum total level of radiation recommended by guidelines, and the upper bound of guideline-based care was set as the number of 180 cGy fractions which would need to be delivered to achieve the maximum total level of radiation recommended by guidelines.

All the providers examined were overseen by HealthHelp during the entire study period. As oversight began in May 2009, by the beginning of the study period in 2011, providers were familiar with HealthHelp procedures. Patients documented as having received 0 fractions of radiation were excluded from the study, and no case rate payments were made for these patients.

For the treatment of breast cancer, care was considered guideline-based hypofractionated care if between 16 and 21 fractions were delivered or guideline-based conventional care if between 25 and 35 fractions were delivered. Patients with breast cancer who received 22–24 fractions were considered to have received guideline-based care, as it is possible that these patients were receiving conventional care but were unable to complete their treatment due to unexpected toxicity or other events. Reclassifying these patients as not receiving guideline-based care was also examined, and found to not impact the conclusions of the study. As hypofractionation is not used by the provider for prostate cancer, it was not analyzed. Hypofractionation for skin cancer was likewise not analyzed as it could not be determined from the claims. The change in the proportion of patients receiving guideline-based care was assessed using unadjusted relative risk. All relative risk scores and 95% confidence intervals were calculated using MedCalc statistical software. [10] Two-tailed values from Student’s two sample t-test assuming unequal variances were used to compare means. The rates of fractionation below the range indicated by clinical guidelines (under-treatment) and above the range indicated by clinical guidelines (over-treatment) were assessed using these techniques as well.

There are trends in treatment patterns that may change over time that are unrelated to payment models, yet there are no references for comparison readily available in the existing literature or in publicly available sources. Accordingly, guideline-based care was reported for two for-profit radiation oncology multi-site providers (one in Florida and one in Arizona) run by different corporations that exclusively held fee-for-service contracts with Humana during the same time periods as a reference for comparison. These providers were referred to as the reference population. No direct comparisons were made between the study and reference population. A difference in differences analysis was not performed, as the reference population may not be adequately comparable. The endpoints were reported for the reference group only to provide contextual evidence where none exists in the public domain.

## Results

A total of 948 patients were included in the study, 433 in 2011 under pure fee-for-service and 515 in 2013 after the introduction of case rate payments. Between 2011 and 2013, there were no significant changes in guideline-based care in breast, lung, and skin cancer **(Table 2)**. However, there were a significantly higher proportion of patients with bone metastases and prostate cancer receiving guideline-based care after the introduction of the case rate, p<0.05. When all cancer types were aggregated, there was no significant change in the delivery of guideline-based (relative risk = 1.0, 95% confidence interval 0.98–1.08, p = 0.20). The mean number of fractions delivered was higher in 2013 for breast, prostate, and skin cancer, but was lower for bone and lung cancer. The mean fractions delivered by modality and cancer type were compared as well, and significant changes (p < .05) were only found for 2D3D in the treatment of bone metastases (14.2 fractions to 11.4 fractions) and breast cancer (27.1 fractions to 32.1 fractions). In the case of IMRT, prostate cancer patients (who were only treated with IMRT by the provider) had their mean fractions increase from 37.4 to 41.1 (p < .05). The subset of patients to whom case rates were applied in 2013 was isolated, and there likewise is not a significantly different rate of delivery of guideline-based care, in aggregate or by cancer type. Chi-squared tests were conducted by cancer type to examine whether the case rate patients had a significantly different change in guideline-based care compared to the non-case rate patients treated by the same provider, and none of the tests were significant.

**Table 2:** Percentage of patients receiving guideline-based care and mean number of fractions delivered.

Cancer Type	Measure	2011 Fee-For-Service	2013 Case Rate	RR	95% CI	P-Value of Change in Mean
**Bone mets.**	* *	** **		2.0†	(1.21–3.24)	
** **	*%*	30.2%	60.0%			
** **	*n*	43	75			
** **	*Mean*	13.7	11.4			0.03†
** **	*±SD*	5.5	5.6			
**Breast**	* *			1.0	(0.94–1.04)	
** **	*%*	95.9%‡	95.1%§			
** **	*n*	123	143			
** **	*Mean*	29.7	30.4			0.31
** **	*±SD*	5.1	6.0			
**Lung**	* *			1.0	(0.89–1.09)	
** **	*%*	91.5%	91.5%			
** **	*n*	59	71			
** **	*Mean*	27.4	27.1			0.84
** **	*±SD*	9.3	9.2			
**Prostate**	* *			1.1†	(1.01–1.15)	
** **	*%*	88.5%	95.2%			
** **	*N*	157	189			
** **	*2D3D mean*	n/a	n/a			n/a
** **	*2D3D ±SD*	n/a	n/a			
** **	*2D3D n*	0	0			
** **	*IMRT mean*	37.4	41.1			0.00†
** **	*IMRT ±SD*	8.7	6.1			
** **	*IMRT n*	157	189			
**Skin**	* *					
** **	*%*	100.0%	91.9%	0.9	(0.84–1.01)	
** **	*n*	51	37			
** **	*2D3D mean*	22.6	24.2			0.20
** **	*2D3D ±SD*	4.0	6.5			
** **	*2D3D n*	50	34			
** **	*IMRT mean*	Redacted	Redacted			Redacted
** **	*IMRT ±SD*	Redacted	Redacted			
** **	*IMRT n*	1	3			
**Overall**	* *			1.0	(0.98–1.08)	
** **	*%*	86.6	89.3			
** **	*n*	433	515*			

Results redacted for groups with n<10.

CI = confidence interval, mets. = metastases, RR = relative risk, SD = standard deviation.

*An intent-to-treat approach was used for this population where case rates were introduced, yet not all patients were covered under the case rate arrangement. Results for 2D3D and IMRT fractionation are displayed separately for prostate and skin cancer due to the differences in the guidelines.

^†^*P* < .05.

^‡^16–21 fractions were delivered to 8% of those who received guideline-based care (9/118).

^§^16–21 fractions were delivered to 10% of those who received guideline-based care (13/136).

The under-treatment rate significantly declined (p = 0.008) from 4% to 0% after the introduction of case rate payments for the aggregate of all cancers. While 10% of patients with prostate cancer and 5% patients with lung cancer were under-treated in 2011, none were in 2013. Over-treatment rates remained steady at 9% in 2011 and 2013, with no significant change (p = 0.20).

In the reference population, which never was exposed to case rate payments, there was no change in the proportion of guideline-based care by cancer type, with the exception of prostate cancer, which increased significantly from 2011 to 2013. Statistics on the level of guideline-based care delivered to the reference population are shown in **Table 3**.

**Table 3:** Percentage of patients receiving guideline-based care and mean number of fractions delivered for the reference population.

Cancer Type	Measure	2011 Fee-For-Service	2013 Fee-For-Service	RR	95% CI	P-Value of Change in Mean
**Bone mets.**	* *			1.1	(0.65–1.79)	
** **	*%*	50.0%	53.8%			
** **	*n*	22	39			
** **	*Mean*	12.1	10.7			0.49
** **	*±SD*	8.4	4.2			
**Breast**	* *			0.9	(0.08–1.02)	
** **	*%*	94.7%	85.9%			
** **	*n*	38	71			
** **	*Mean*	26.6	27.5			0.57
** **	*±SD*	7.9	8.3			
**Lung**	* *			0.9	(0.76–1.17)	
** **	*%*	87.5%	82.4%			
** **	*n*	24	34			
** **	*Mean*	24.3	25.6			0.65
** **	*±SD*	10.8	11			
**Prostate**	* *			1.5†	(1.25–1.77)	
** **	*%*	58.5%	87.1%			
** **	*n*	106	124			
** **	*2D3D mean*	36.2	38.7			0.51
** **	*2D3D ±SD*	8.8	11.3			
** **	*2D3D n*	71	10			
** **	*IMRT mean*	36.8	38.6			0.25
** **	*IMRT ±SD*	8.2	7.1			
** **	*IMRT n*	35	114			
**Skin**	* *			1.1	(0.95–1.15)	
** **	*%*	91.9%	97.5%			
** **	*n*	37	40			
** **	*2D3D mean*	18.3	20.8			0.14
** **	*2D3D ±SD*	7.4	7.4			
** **	*2D3D n*	37	40			
** **	*IMRT mean*	n/a	n/a			n/a
** **	*IMRT ±SD*	n/a	n/a			
** **	*IMRT n*	n = 0	n = 0			
**Overall**	* *			1.2†	(1.05–1.29)	
** **	*%*	72.2%	83.4%			
** **	*n*	227	308			

CI = confidence interval, mets. = metastases, RR = relative risk, SD = standard deviation.

^†^*P* < .05.

## Discussion

These findings suggest that the introduction of case rate payments did not adversely affect the rate of guideline-based care at the provider examined. However, the bone metastases and prostate cancer subpopulations experienced a significant increase in the rate of guideline-based care after the introduction of case rates. While there is no previously published literature assessing the impact of case rate payments in radiation oncology, there are studies assessing reimbursement and payment models in medical oncology. [11–14] Two prior studies have shown that reductions in reimbursement for chemotherapy impact prescribing patterns. [11–12] Building upon these findings, an evaluation of switching reimbursement from being for chemotherapy drugs to being for cognitive services showed an impact on prescribing behavior. [13] Finally, case rate payments in medical oncology, coupled with incentives for quality and the elimination of financial incentives for the use of chemotherapy drugs, have been associated with increased chemotherapy spending and reduced medical spending. [14]

While the prior findings are important, they are limited to medical oncology and extrapolation to radiation oncology may be difficult. Furthermore, it is arguably more important to assess the impact of payments on guideline-based care, rather than changes in patterns from baseline. Accordingly, this is the first study to date with evidence to suggest that the introduction of case rate payments in radiation oncology does not adversely impact guideline-based care. Guideline-based care is an indicator of quality improvement, as standards organizations have developed guidelines based upon prior research on the relationship between treatment and clinical outcomes.

The rates of guideline-based care specified in this study may have been impacted by confounding variables. While an attempt was made to control for the nature of the patient population by examining the same providers in 2011 and 2013, it is possible that there were shifts in the patient populations or practice patterns due to other factors. To further account for this, a reference population that used only fee-for-service reimbursement during the same period was examined. Both the study and the reference providers had significantly higher rates of guideline-based care for prostate cancer in 2013, suggesting that there may have been external changes affecting prostate cancer care during this time period. The increasing attention to guideline-based care in radiation oncology may have led to global improvements in care over time, regardless of the payment methodology.

It is possible that patient-level variation in health plan products may have impacted the findings. This study employed an intent-to-treat approach and included all patients in the payer’s database, regardless of product type. This is most reflective of clinical practice where clinicians treat patients with a mix of insurance types and payment methods without awareness of the arrangements of each individual patient, although it is possible that a for-profit provider might have an increased level of awareness of patients’ payment arrangements. Furthermore, the study did not control for the percentage of patients treated by each clinic under case-rate payments, as the number of non-Humana patients being treated by the clinic is unknown. It is possible that case rate payments do not impact clinical practice until they constitute a certain proportion of a provider’s patients.

Misclassification bias in this study was possible since circumstances not apparent from the claims data may have justified the treatment regimens which were given. Clinical data were not available to explore misclassification for 63% of the 2011 cases and 51% of the 2013 cases, as the cases were either exempt from the RBM process or circumvented it. As a result of the lack of clinical data on cases that did not go through the RBM, it was not possible to distinguish which patients received palliative and curative care. The misclassification bias may have been particularly pronounced in patients with breast cancer or bone metastases. For breast cancer patients, it was not possible to discern between conventional or hypofractionated regimens from claims data alone. Among the breast cancer patients that received guideline-based care, 16–21 fractions were delivered to 8% of the 118 patients in 2011 and 10% of the 136 patients in 2013, suggesting that there has been slowly increasing adoption of hypofractionation, consistent with the literature. [15–16] However, patients scored as having been properly hypofractionated may have been patients who were receiving a conventional regimen but received an inadequate number of fractions. Likewise, patients scored as having been properly fractionated under a traditional regimen may have been on a hypofractionated regimen and received an excessive number of fractions. For bone metastases, a uniform set of guidelines was applied to all patients, rather than separate guidelines for patients with axial versus peripheral metastases. As the guidelines used for bone metastases were the more liberal of the two, this biased the findings towards guideline-based care. Likewise, patient demographics were not used to determine which set of guidelines should be used, in the event that guidelines were linked to demographic factors. This limitation led to wide ranges of fractions being classified as guideline-based care, limiting the ability of the study to detect subtle changes in guideline-based care. These biases increased the likelihood that patients in all conditions of the study would be scored as having received guideline-based care. Consistent with the trend towards more evidence-based treatment, the proportion of bone metastases patients receiving over 10 fractions decreased from 70% in 2011 to 40% in 2013.

The study reflects the experiences of a single multi-site radiation oncology provider, and thus may not be generalizable to other providers. Furthermore, the sample size may be inadequate to detect subtle treatment effects. It was not possible to increase the sample size, as a complete set of claims from the provider was used.

Further investigation is needed to isolate the impact of the payment methodology. It is possible that the dissemination of the guidelines into the marketplace may have impacted both the reference and study populations, accounting for some of the improvements that both experienced. From the data on the reference population, it appears that the treatment of prostate cancer during the study period may have shifted towards being more congruent with guidelines.

## Conclusions

After the introduction of case rate payments in this radiation oncology multi-site provider, there were no significant changes in guideline-based care for breast, lung or skin cancer, however, guideline-based care improved in both bone metastases and prostate cancers. While these findings are the first to investigate the impact on clinical practice when case rates are in employed in radiation oncology, further investigation is needed to isolate the impact of the payment model, replicate the findings in other populations and practice settings, and assess a broader set of outcomes. As providers and payers continue to integrate evidence-based practice changes and explore new payment models, they must collaboratively assess the impact on quality of care, value, member experience, and other outcomes. These findings suggest that there is additional value in studying case rate payments in radiation oncology.
